# Translational Potential of Fluorescent PARP1 Inhibitor as a Molecular Contrast Agent for Diagnosis of Basal Cell Carcinoma

**DOI:** 10.2967/jnumed.124.269428

**Published:** 2025-08

**Authors:** Miriam Ricanati, Ucalene Harris, Neil M. Neumann, Banu Farabi, Nicholas Kurtansky, Anthony Rossi, Chih-Shan J. Chen, Melissa P. Pulitzer, Milind Rajadhyaksha, Stephen Dusza, Ashish Dhir, Manu Jain

**Affiliations:** 1 Dermatology Service, Memorial Sloan Kettering Cancer Center, New York, New York;; 2 Department of Pathology, Memorial Sloan Kettering Cancer Center, New York, New York;; 3 Department of Dermatology, New York Medical College, Valhalla, New York; and; 4 Office of Entrepreneurship and Commercialization, Memorial Sloan Kettering Cancer Center, New York, New York

**Keywords:** PARPi-FL topical safety, translational potential, basal cell carcinoma, benign lesions, SKH1-Hr^hr^ mice, Yorkshire pigs

## Abstract

Basal cell carcinoma (BCC), the most common skin cancer, typically requires biopsy for definitive diagnosis. Reflectance confocal microscopy is a noninvasive rule-out test, but the lack of nuclear contrast in BCC often leads to missed diagnoses. Our previous research in ex vivo human skin indicated that PARPi-FL, a poly(adenosine diphosphate ribose) polymerase 1 (PARP1) inhibitor–targeted fluorescent contrast agent, penetrated intact skin when applied topically and improved the diagnosis of BCC compared with reflectance confocal microscopy alone. This study refined PARPi-FL’s concentration and application timing to assess its detectability, safety, and feasibility for topical use in clinics and investigated its potential to distinguish BCC from nonmalignant clinical mimickers. **Methods:** We assessed PARP1 expression in 86 BCCs and 76 nonmalignant mimickers via immunohistochemistry. PARPi-FL (10 μM) permeability on ex vivo human skin was evaluated using a fluorescent confocal microscope (FCM) after 5 min compared with the previously evaluated times of 10–30 min. We evaluated the detectability of PARPi-FL’s fluorescent signal (10 μM; 5 and 30 min) via FCM through intact skin in excised human BCC tumors and compared it with the signal in 4 benign lesions. FCM imaging was conducted in anesthetized tumor-bearing B6 K5-Gli2 mice to evaluate topical application with gauze, simulating in vivo human imaging. Quantitative measurements of FCM signal intensities were taken with varying PARPi-FL concentrations (>10 μM) at 5 min. Systemic and cutaneous toxicity were assessed in SKH1-Hr^hr^ mice and Yorkshire pigs. **Results:** PARP1 was overexpressed in BCC lesions compared with nonmalignant lesions. PARPi-FL penetrated intact human skin and labeled dermal structures within 2–5 min. A strong fluorescent signal from BCC lesions was detectable through the skin surface to a depth of approximately 100 µm after 5 min, whereas benign lesions showed lower and more variable signals. PARPi-FL application using gauze proved effective, with detectable signals in tumor-bearing mice. Increasing the concentration and reducing application time enhanced the nuclear fluorescent signal. No serious toxicity was observed in preclinical species. **Conclusion:** PARPi-FL is a fluorescent molecular contrast agent that has shown preclinical safety and translational potential for use in topical application for noninvasive BCC diagnosis. Its diagnostic accuracy and safety in humans require validation in clinical trials.

## Visual Abstract

**Figure:**
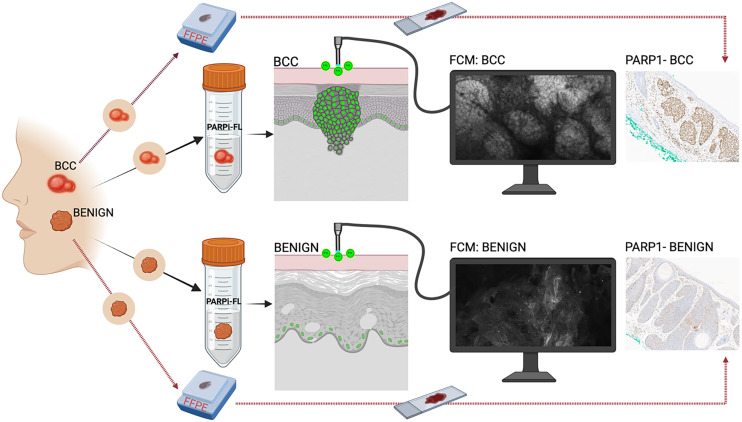

Basal cell carcinomas (BCCs) are the most common cancer in the United States, with approximately 4.3 million cases annually (1). Although most BCCs are nonaggressive (i.e., superficial or nodular), 10%–15% can be locoregionally aggressive and rarely metastatic (2). Diagnosis typically involves clinical inspection, dermoscopy, and biopsy for histopathologic confirmation. Dermoscopy has low specificity (∼50%) and varies by lesion type (pigmented or nonpigmented) and clinician expertise (3), leading to potential misdiagnoses and unnecessary biopsies (4). Although a gold standard, biopsy is invasive, can be psychologically traumatic (5), and may not be necessary for some early-detected BCCs treated nonsurgically when diagnosed correctly in vivo (6).

Although reflectance confocal microscopy has improved the accuracy of skin cancer diagnoses (7,8), the diagnosis of nonpigmented BCCs (∼95% of cases) remains challenging due to the lack of endogenous contrast from melanin (9–11). PARPi-FL, a poly(adenosine diphosphate ribose) polymerase 1 (PARP1) inhibitor–targeted fluorescent contrast agent, is derived from olaparib (Supplemental Fig. 1) (supplemental materials are available at http://jnm.snmjournals.org). It is conjugated to fluorescent boron dipyrromethene dye (Molecular Probes) (12) and binds to PARP1 (a nuclear DNA repair enzyme), which is overexpressed in several malignancies, including melanomas (13–18). PARPi-FL is highly stable, with a low molecular weight suitable for topical application (19), and is being evaluated in a phase 1/2 clinical trial at Memorial Sloan Kettering Cancer Center for in vivo detection of oral squamous cell carcinoma (NCT03085147) (20).

We previously demonstrated that PARP1 is overexpressed in BCCs (18). A 10-μM PARPi-FL solution can passively penetrate ex vivo human and in vivo pig skin after topical administration for 10–30 min. PARPi-FL enhanced BCC visualization in ex vivo human tissues, improving diagnostic accuracy compared with reflectance confocal microscopy images, making it a promising nuclear contrast agent for improving diagnostic accuracy for BCC.

In this study, we aimed to determine if PARP1 would be overexpressed in BCC lesions compared with nonmalignant clinical mimickers and whether PARPi-FL would penetrate skin in less than 10 min and demonstrate fluorescent signal detectability through intact skin in BCC and benign lesions using a fluorescent confocal microscope (FCM). We also evaluated PARPi-FL’s feasibility for use in vivo imaging, safety for local (skin) and systemic toxicity after its topical application, and ability to enhance signal intensity at concentrations exceeding 10 μM with shorter application times.

## MATERIALS AND METHODS

### Patient Population

Memorial Sloan Kettering Cancer Center’s institutional review board approved this study, and all subjects gave written informed consent.

All animal experiments were performed in accordance with the National Institutes of Health’s guide for the care and use of laboratory animals following protocols approved by Memorial Sloan Kettering Cancer Center’s Institutional Animal Care and Use Committee.

### PARP-1 Expression in BCC Versus Nonmalignant Clinical Mimickers in Ex Vivo Human Tissue

Two formalin-fixed, paraffin-embedded tissue sections were cut from 162 formalin-fixed, paraffin-embedded blocks (53.1% BCCs and 46.9% nonmalignant clinical mimickers) and stained with hematoxylin and eosin (H&E) and PARP1 immunohistochemistry. Immunohistochemistry-stained slides were analyzed quantitatively for PARP1 expression (supplemental materials 1) (18). Descriptive statistics and linear regression were used to compare the mean proportions across diagnostic groupings (Supplemental Table 1). Benign lesions were compared with BCC, premalignant actinic keratosis, and inflammatory lesions (group 1); benign and inflammatory lesions were combined and compared with the other categories (group 2).

### PARPi-FL Permeability Determination Using Benchtop Ex Vivo Confocal Microscope (EVCM)

Fresh abdominal skin from 2 patients undergoing reconstructive breast surgery was obtained and cut into 2.5 × 2.5 cm^2^ pieces. Gauze (2 × 2 cm^2^) soaked in 10 μM PARPi-FL or 30% polyethylene glycol phosphate-buffered solution (PEG-PBS) was applied for 5 min (3 tissues with PARPi-FL and 1 tissue with PEG-PBS) or 30 min (2 tissues with PARPi-FL and 1 tissue with PEG-PBS). In one tissue, a 2-min application of PARPi-FL was tested (supplemental materials 2). The occlusion method (18) ensured effective dye delivery. A 0.5 × 1 cm^2^ tissue strip was cut, placed laterally on a slide, and imaged with an EVCM (Vivascope 2500; Caliber Imaging and Diagnostics) in fluorescent mode (488 nm). Tissues were frozen for histopathology.

FCM images were qualitatively analyzed to detect fluorescent signals in the nuclei of epidermis and adnexal structures. FCM results were validated against H&E images (supplemental materials 2).

### PARPi-FL Signal Detectability Through Intact Skin Using In Vivo FCM

Fresh human BCC tissues with intact skin from Mohs surgery were immersed in 10-μM PARPi-FL for 30 (*n* = 3) or 5 (*n* = 1) min and washed with 30% PEG-PBS. Four fresh benign specimens (acrochordon, seborrheic keratosis, sebaceous hyperplasia, and solar lentigo) were collected and immersed in 10-μM PARPi-FL for 5 min. Tissues were imaged through intact skin using FCM, followed by formalin fixation for histopathology. Morphologic features on FCM were validated with H&E-stained images. FCM images from BCC lesions immersed in PARPi-FL for 30 min were qualitatively compared with BCC and benign lesions immersed in PARPi-FL for 5 min (supplemental materials).

### Feasibility of Topical Application of PARPi-FL Using Gauze and In Vivo FCM Imaging

In vivo FCM imaging involved 5 female B6 K5-Gli2 transgenic mice (10 tumor sites; 2 tumors per mouse; sourced from Department of Dermatology, University of Michigan, and bred at MSK) with BCC and 3 age- and sex-matched controls (2 normal sites/mouse; Jackson Laboratory). Gauze soaked with 1 or 5 μM of PARPi-FL or 30% PEG-PBS was applied to tumors (3–5 mm) on earlobes and contralateral normal earlobes for 15 or 30 min (supplemental materials 4). Sites were washed and imaged after application, followed by biopsy.

Biopsied lesions were fixed in formalin and sectioned for H&E and PARP1 immunohistochemistry staining. Immunohistochemistry-stained slides were scanned, annotated, and analyzed (supplemental materials 1).

### FCM Imaging and Analysis

An FCM (ViewnVivo; Optiscan) with a 488-nm laser was used for imaging human ex vivo BCC lesions and in vivo mice through intact skin. Z-stacks were captured from the skin surface to the superficial dermis to assess skin layers and signal depth. Epidermis and BCC tumor nodules were identified by morphology. FCM images were visually evaluated for variations in fluorescence intensity in BCC lesions across different concentrations and time points. FCM results were validated against H&E-stained images (supplemental materials 3).

### In Vivo Toxicology Studies

#### Mice

An extended single-application toxicology study was conducted with 24 female SKH1-Hr^hr^ hairless albino mice (Charles River Laboratories) randomly assigned to 4 groups (*n* = 6 per group). Mice received a 30-min application of 30% PEG-PBS or PARPi-FL (7.5, 20, or 100 μM) on the flank using 2 × 2 cm^2^ gauze (Supplemental Fig. 2). Clinical evaluations were conducted before and after application. Three mice per group were sacrificed at 24 h to assess acute effects and the remaining 3 at 14 d to evaluate delayed effects. Body weight and clinical signs were monitored daily. Blood samples were collected at 24 h and 2 wk for complete blood count with differential and comprehensive metabolic panel assessments, followed by necropsy and histopathology of the skin and liver (21) (supplemental materials).

#### Pigs

Three adult female Yorkshire pigs were used to assess acute cutaneous toxicity of topical PARPi-FL. One milliliter of PARPi-FL (10 μM) or saline (5 sites per pig) was dispensed into wells attached to the skin with a 30-min dwell time (18). Clinical and dermoscopy images were taken before and after application, followed by skin biopsies. Two dermatologists and 2 dermatopathologists, masked to treatment, evaluated images and H&E-stained slides for allergic contact dermatitis. Adjusted odds ratios were calculated to quantify associations between dermoscopy feature presence and assignment (supplemental materials 6).

### Quantification of PARPi-FL Signal at Higher Concentrations with Shorter Application Times

Fresh, normal human abdominal skin obtained from patients undergoing plastic surgery was cut into 2.5 × 2.5 cm^2^ pieces. Gauze soaked with PARPi-FL (50, 70, and 100 μM) was topically applied on each piece for 5 min. The comparator was a 10-μM PARPi-FL sample (*n* = 1) with a 30-min dwell time. Vertical sections were prepared and imaged with an LSM880 FCM. FCM images were quantitively analyzed for nuclear signal (supplemental materials 2 and 7).

## RESULTS

### PARPi-FL’s Potential for Differentiating BCC from Nonmalignant Clinical Mimickers

**FIGURE 1.**
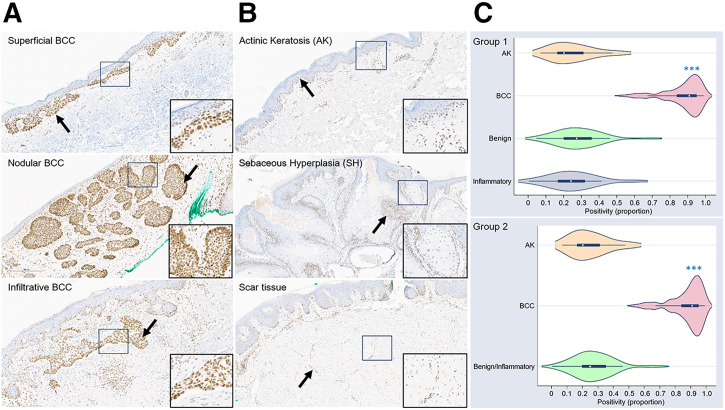
PARP1 overexpression in BCCs (A) vs. nonmalignant mimickers (B); insets and arrows show nuclear positivity. (C) Violin plots of positivity by diagnostic groups. Immunohistochemistry images, ×10. ***P < 0.001 compared with benign or benign/inflammatory lesions.

In group 1, the mean PARP1 proportion of positivity ranged from 0.248 ± 0.124 in actinic keratosis lesions (premalignant) to 0.877 ± 0.107 in BCC lesions. BCC lesions showed significantly higher positivity than did benign lesions (*P* < 0.001). No significant differences were found between benign lesions and actinic keratosis (*P* = 0.338) or inflammatory (*P* = 0.405) lesions. Similar results were observed in group 2 when combining benign and inflammatory lesions as a reference group (Supplemental Table 1). BCC lesions exhibited consistent nuclear staining (Fig. 1A), with some loss in necrotic areas, consistent with findings from our previous study (18). Nuclear positivity was observed in the basal cell layer of non-BCC lesions (Fig. 1B). Demographic and lesion details appear in Supplemental Table 2.

### PARPi-FL Showed Rapid Permeability in Ex Vivo Human Skin

**FIGURE 2.**
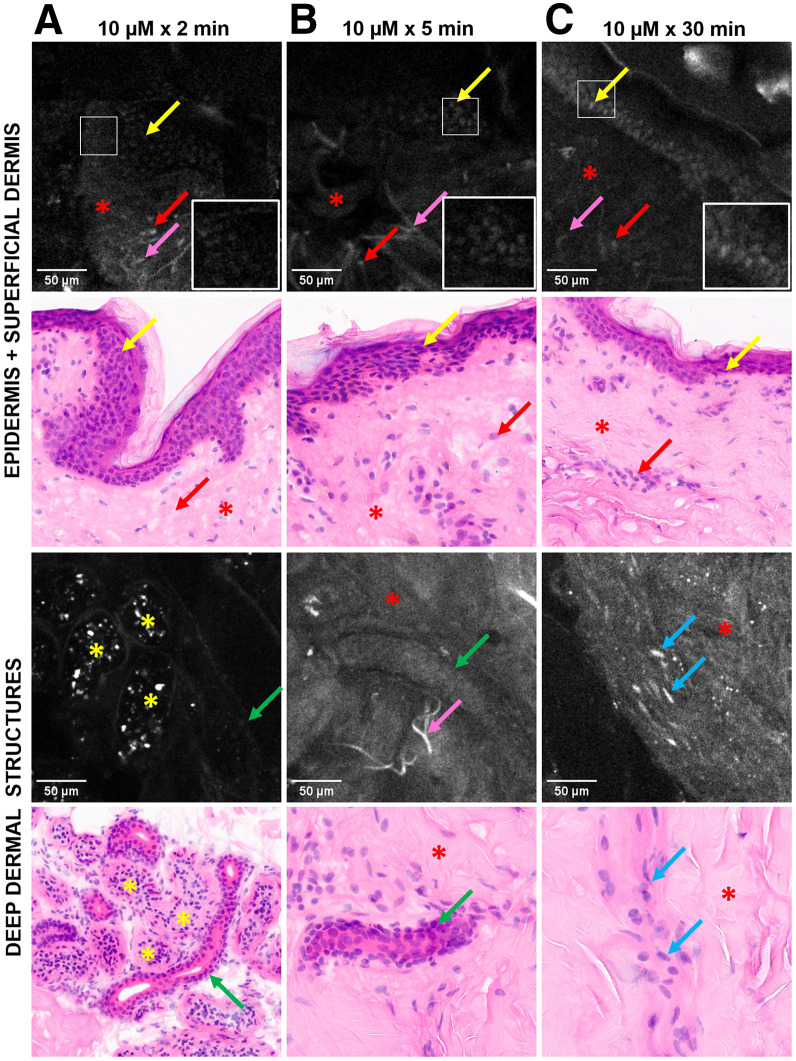
PARPi-FL (10 μM) rapidly penetrates intact human skin. FCM images acquired with EVCM device show nuclear labeling in basal cells (yellow arrows), eccrine duct lining (green arrows), muscle fibers (blue arrows), and inflammatory cells (red arrows) at 2 (A), 5 (B), and 30 (C) min. Nonspecific signals are shown in eccrine gland secretions (yellow asterisks), elastin fibers (pink arrows), and collagen (red asterisks). Corresponding H&E-stained images, ×30. Elastin fibers are not readily visible with H&E staining.

No nuclear signal was detected by EVCM in tissues treated with 30% PEG-PBS. Topical application of 10-μM PARPi-FL resulted in detectable fluorescence in the nuclei of basal epidermal cells and dermal structures (hair follicles, eccrine ducts, muscle fibers, inflammatory cells) as early as 2 and 5 min (Fig. 2). The depth of signal detectability observed depended on the location of adnexal structures in the dermis. The depth of adnexal structures and nuclear sizes measured by EVCM were comparable to those in H&E images, albeit a limited sample (Supplemental Table 3). Nonspecific (nonnuclear) binding was observed in dermal collagen, elastin, and eccrine gland secretions (Fig. 2).

### PARPi-FL Signal Detection Through Intact Skin Using In Vivo FCM

#### Fresh BCC Human Tissue

**FIGURE 3.**
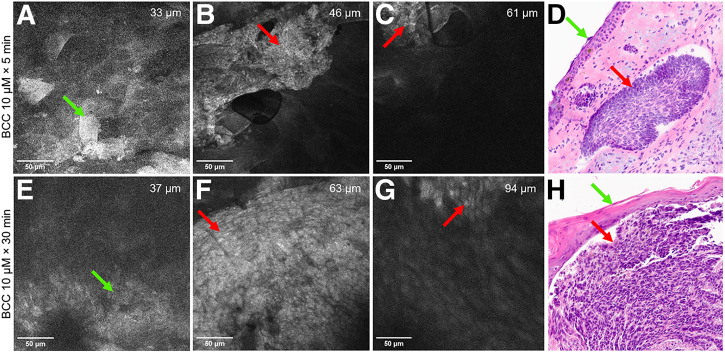
PARPi-FL-labeled (10 μM) FCM signal at 5 (A–C) and 30 (E–G) min in excised human BCC lesions through intact skin. Nonspecific binding to corneocytes (A and E; green arrows), strong signal in tumor nuclei (B and F; red arrows), and diminishing signal intensity in tumor nuclei in dermis (C and G; red arrows). H&E-stained images of nodular BCC (D and H) showing tumor nodules (red arrows) and overlying epidermis (green arrows), ×30.

Fluorescent signal within BCC tumor nodules was detectable as bright, round-to-oval clusters with crowded nuclei (Figs. 3B and 3F). Nodule depth ranged from 43 to 94 µm, depending on the location within the dermis (Figs. 3B, 3C, 3F, and 3G). BCC nuclear signals were visible at 5 min, comparable to those observed after 30 min of application (Figs. 3A–3C and Figs. 3E–3G). Nonspecific binding was noted in corneocytes (Figs. 3A and 3E) within a 40-µm depth from the skin surface. BCC tumor depth from the skin surface, epidermal thickness, corneocyte size, and nuclear size were comparable to that found in H&E-stained tissues, albeit in a limited sample (Supplemental Table 4). H&E staining confirmed BCC diagnosis and identified subtypes (nodular/superficial or mixed) (Figs. 3D and 3H).

#### PARPi-FL Demonstrated Lower and Variable Nuclear Intensity in Benign Lesions

**FIGURE 4.**
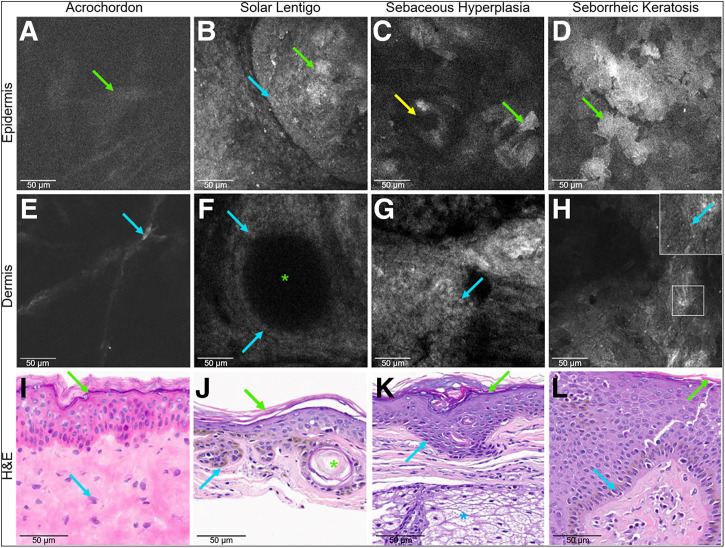
PARPi-FL (10 μM) binding in fresh benign lesions after 5 min. Epidermis (A–D) shows nonspecific binding to corneocytes (green arrows). Low-intensity nuclear signals (blue arrows and inset): fibroblast in acrochordon (E), basal cells in solar lentigo (F), sebaceous hyperplasia (G), and seborrheic keratosis (H). Hair follicle (C; yellow arrow); corneal cyst (F and J; green asterisk); sebaceous gland (K; blue asterisk) not visible. H&E images (I–L), ×30.

BCC lesions exhibited an intense and nearly uniform PARPi-FL signal (Fig. 3B), whereas benign lesions displayed a lower, more variable signal, mostly restricted to the nuclei of the basal epidermal cells (Fig. 4). Nonspecific binding could be observed in corneocytes and the superficial spinosum cells in all lesions (Figs. 4A–4D).

FCM signals were compared with the H&E-stained images. Acrochordons displayed sparse elongated nuclei via FCM (Fig. 4E), corresponding to fibroblasts on H&E-stained images (Fig. 4I). Solar lentigo displayed dark round structures (Fig. 4F) at the dermoepidermal junction, lined by small nuclei (Fig. 4F), which matched corneal cysts and basal nuclei (Fig. 4J). Sebaceous hyperplasia showed a sheet of fluorescent nuclei (Fig. 4G), corresponding to basal cell nuclei (Fig. 4K). Sebaceous glands were not identifiable on FCM imaging. In seborrheic keratosis, bright big polygonal cells were identified in the top layers (Fig. 4D), with a sheet of small nuclei at the dermoepidermal junction (Fig. 4H), corresponding to hyperkeratosis and basal cell nuclei on H&E-stained images (Fig. 4L).

Epidermal thickness and depth of basal layer (from the surface) and basal nuclear and corneocyte size on FCM were comparable with H&E-stained images, albeit in a limited sample (Supplemental Tables 5 and 6).

#### Mice BCC Tumor Detection In Vivo

**FIGURE 5.**
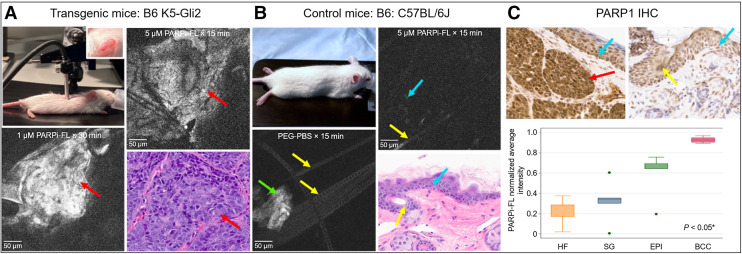
PARPi-FL signal in vivo in mice using FCM. (A) FCM probe on anesthetized transgenic mice; (inset) FCM probe on earlobe tumor cluster. PARPi-FL–labeled BCC (red arrows) shows bright nuclear signal, corresponding to nodular BCC on H&E-stained image. (B) Anesthetized control mice show low nuclear signal (blue arrow) in PARPi-FL–treated normal earlobes, with no signal in site treated with PEG-PBS and corresponding H&E-stained image. Corneocyte (green arrow) shows nonspecific binding. (C) PARP1 expression in BCC (red arrow) compared with normal skin: basal epidermal nuclei (blue arrows) and nuclei of hair follicles (HF; yellow arrows). Boxplot shows higher PARP1 levels in BCC vs. HF, sebaceous glands (SG), and epidermis (EPI). Dots represent observations outside 5th and 95th percentiles for each category. Results were compared using 1-way ANOVA (*P < 0.05) followed by post hoc test (significant levels in text). H&E-stained and immunohistochemistry (IHC) images, ×40.

When applied topically to BCC tumors in mice, 5-μM PARPi-FL (15 min) showed an FCM signal similar to that of 1-μM PARPi-FL for 30 min; the signal was visible 70–100 µm from the skin’s surface (Fig. 5A). The intensity of the PARPi-FL signal in the epidermal basal nuclei of the adjacent tumor-free earlobe and in control mice was lower than that in tumor sites (Fig. 5B). No signal was observed in sites treated with 30% PEG-PBS. BCC presence was confirmed on H&E-stained images (Fig. 5A). Epidermal thickness, corneocyte size, tumor depth, and nuclear size were comparable between FCM and H&E-stained images, albeit in a small sample size (Supplemental Table 7). One-way ANOVA, followed by Bonferroni post hoc test, indicated overexpression of PARP1 in BCC lesions compared with normal surrounding skin structures, such as the epidermis (*P* = 0.035), sebaceous glands (*P* < 0.001), and hair follicles (*P* < 0.001) (Fig. 5C).

### Topical Application of PARPi-FL in SKH1-Hr^hr^ Hairless Albino Mice Did Not Cause Acute or Delayed Adverse Effects

No signs of clinical toxicity were observed in mice after a single topical application of PEG-PBS or PARPi-FL at various concentrations. Animals maintained normal body weight, survived to study endpoints, and showed no skin toxicity or allergic contact dermatitis at 30 min, 24 h, or 14 d. Histopathologic examination at application sites showed no signs of allergic contact dermatitis at 24-h and 2-wk time points (Supplemental Figs. 3 and 4). Complete blood count revealed no immediate adverse effects, except for 1 animal in the highest-dose group (100 μM) who exhibited slightly reduced platelet (89 K/µL) and reticulocyte (173.60 K/µL) counts at 24 h (data of individual animals not shown). No such effects were observed in the 14-d group, indicating potential reversibility. Aspartate aminotransferase levels increased slightly at 24 h and 2 wk with PARPi-FL compared with PEG-PBS (Supplemental Table 8). However, this effect may be unrelated to PARPi-FL, as 1 animal in the 24-h group and another in the 14-d group of the control group also exhibited elevated aspartate aminotransferase levels. One animal in the highest-dose group had elevated creatinine kinase (2,303.9 U/L) at 24 h, which was not observed in the 14-d group. Levels of other enzymes, alanine aminotransferase, alkaline phosphatase, and total bilirubin remained unchanged. Liver histopathology at 14 d showed no drug-induced injury (Supplemental Fig. 5).

### Topical Application of PARPi-FL in Pigs Did Not Result in Acute or Delayed Skin Toxicity

**FIGURE 6.**
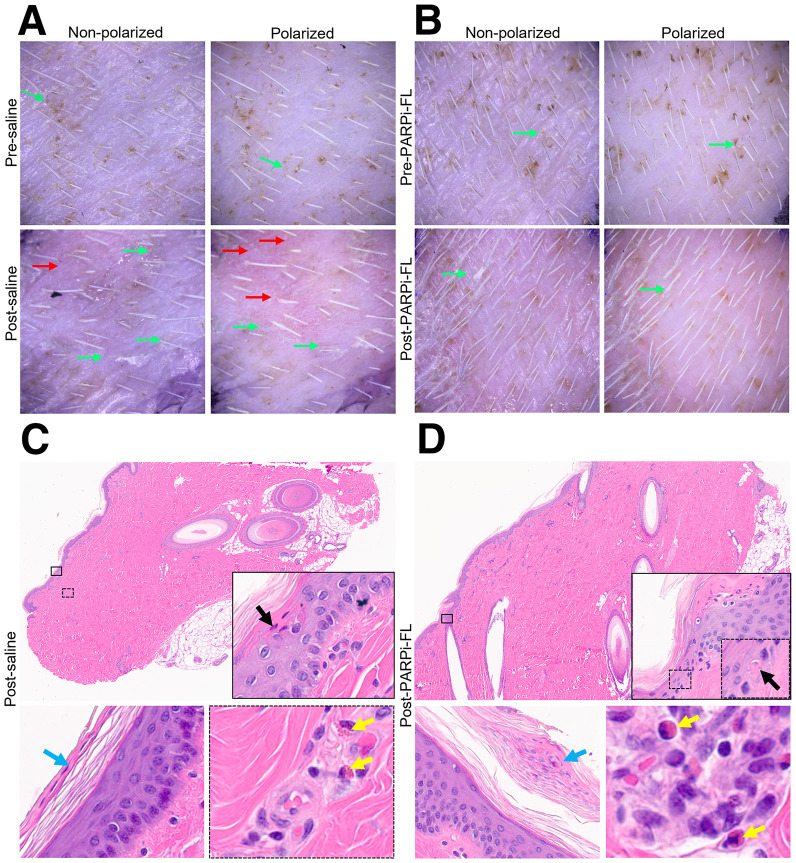
Features of allergic contact dermatitis in pig skin treated with 10-μM PARPi-FL (30 min) or saline. (A and B) Dermoscopy images show scaling (green arrows) and erythema (red arrows). (C and D) H&E-stained images reveal necrotic cells (black arrows and insets), eosinophils (yellow arrows and insets), and parakeratosis (blue arrows; image from skin of another pig). H&E-stained images, ×4 and ×40.

Signs of dryness, edema, erythema, scaling, and vesicles were 7%–30% prevalent on dermoscopy in pigs before applying saline (Fig. 6A) or PARPi-FL (Fig. 6B). After application, the prevalence of these signs increased by 10%–43%. After the application of PARPi-FL, the prevalence of bullae, edema, scaling, and vesicles was higher (30%, 60%, 60%, and 30%, respectively) compared with rates observed after the application of saline (21%, 36%, 29%, and 21%, respectively). Although not powered to detect statistically significant differences, we calculated *P* values for the incidence of dermoscopy parameters, revealing significance for scaling only (*P* = 0.002; Supplemental Tables 9 and 10).

Most histologic toxicity features of contact dermatitis were absent in the saline-treated group (Fig. 6C), except for necrotic keratinocytes, parakeratosis, and vacuoles alteration, despite the small sample size (*n* = 4). Mild toxicity was more common with PARPi-FL application (Fig. 6D), but no samples exhibited moderate or severe toxicity. No statistically significant differences were observed between PARPi-FL and saline application in histopathologic changes related to contact dermatitis (Supplemental Table 11).

### High Concentrations of PARPi-FL Applied at Shorter Time Points Produced Enhanced FCM Signals in Ex Vivo Normal Human Skin

**FIGURE 7.**
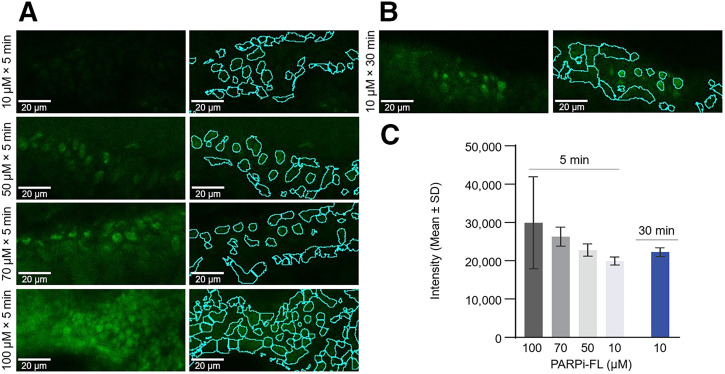
Effect of high PARPi-FL concentration and reduced application time on nuclear signal intensity. FCM images with nuclear signal at dermoepidermal junction (left panels) and annotated region of interest (nuclei) after 5 (A) and 30 (B) min of PARPi-FL topical application (*n* = 1 per concentration). (C) Quantitative FCM intensity at different concentrations and application times.

Fluorescence intensity was positively associated with increasing dye concentrations (50–100 μM) using a shorter application time (5 min) compared with 10 μM at 30 min (Fig. 7; Supplemental Table 12).

## DISCUSSION

We previously identified PARPi-FL as a promising fluorescent nuclear contrast agent for BCC detection (18). We demonstrated PARP1 overexpression in BCCs and PARPi-FL’s ability to passively permeate intact skin within 10–30 min of topical application and improve the diagnostic accuracy of BCC in ex vivo human tissues compared with reflectance confocal microscopy, demonstrating that 10-μM PARPi-FL was optimal for permeability (18). The current study explored varying concentrations and application times in preclinical settings to assess PARPi-FL’s translational potential and safety when used in the clinical setting. We found that a 10-μM PARPi-FL solution can penetrate intact skin in 2–5 min and label nuclei in the dermoepidermal junction and adnexal structures. Imaging speed can determine the successful clinical integration of a novel technology.

Key to clinical translation is the ability to differentiate BCC lesions from nonmalignant mimickers. We demonstrated significantly higher PARP1 expression in BCC lesions compared with benign, precancerous (actinic keratosis), and inflammatory lesions, which would result in lower PARPi-FL binding and signaling in the latter compared with BCC lesions in vivo. Our results align with prior findings demonstrating elevated PARP1 expression in human tumor types (15).

We could detect PARPi-FL signal in the nuclei of BCC nodules and benign lesions to a depth of 100 µm. Signals were more uniform and intense in BCC lesions compared with benign lesions. We described, for the first time (to our knowledge), morphologic features in these lesions that could aid in their differential diagnosis at the bedside. Our PARPi-FL FCM-imaging approach could improve diagnostic accuracy of BCC and reduce unnecessary biopsies.

A main limitation of this study was the small sample size of fresh lesions, which restricted our ability to fully assess PARPi-FL’s diagnostic significance. Ex vivo specimens lack blood circulation in the excised tissue, which may have affected signal detection. However, microcirculation in vivo may aid in dye clearance (22) and impart better tumor visualization. We did not topically apply PARPi-FL-soaked gauze on BCC tissues because of the small tissue size (<5 mm).

To simulate in vivo BCC imaging, we performed live imaging in transgenic mice with BCC (23). This approach demonstrated the feasibility of topical application and FCM imaging for clinical trials. However, we used 5-μM PARPi-FL instead of 10-μM PARPi-FL in mice, as the higher concentration caused signal saturation because of their thinner skin. While mouse skin is thinner than human skin, most BCC lesions grow on facial skin, which is comparable in thickness to mouse skin. Although the thickness of pig skin is similar to that of humans, pigs do not develop BCC lesions (24).

The FCM used for imaging lesions may not be ideal for in vivo imaging in humans due to its slower imaging speed (1 frame/s), small field of view (475 µm × 475 µm), and ability to image only in fluorescent mode. Relying on nuclear signal for BCC differentiation may be limiting; an ideal diagnosis should incorporate cytologic and architectural details (25). Future advancement should include a faster device with a larger imaging field combining reflectance and fluorescence modes, which could be digitally colorized to enhance diagnostic accuracy (7).

As the first investigators (to our knowledge) to perform ex vivo and in vivo imaging of these lesions with PARPi-FL, we validated our results using H&E-stained images. Quantitative assessments were comparable between the modalities. Results were limited due to small sample size.

PARPi-FL is being evaluated in a phase 1/2 trial for detecting oral squamous cell carcinoma, though application time in the oral cavity is 2 min. Because this is a new administration route, we investigated safety with application times extended to 30 min in preclinical species and found no major adverse effects (17). Our preclinical study confirmed safety at 100 times the expected clinical dose, suggesting single-dose topical application will be well tolerated in humans. SKH1-Hr^hr^ mice are ideal for evaluating topical applications, toxicity, and immune responses (24).

After confirming safety at higher concentrations, we assessed higher concentrations of PARPi-FL in excised human tissues with shorter application times. Our quantitative analysis revealed that a higher concentration (>10 μM) significantly enhanced the FCM signal with an application time of 5 min versus 30 min. This finding could expedite in vivo imaging and improve BCC detection in humans. Although this study focused on BCC and its benign mimickers, PARPi-FL expression in melanoma and SCC should be explored.

## CONCLUSION

This study provides a framework for a clinical trial to establish PARPi-FL as a topical contrast agent for detecting BCC in vivo in humans. Our findings highlight the translational potential of this approach, including its safety in preclinical species, to advance noninvasive BCC diagnosis and reduce unnecessary benign biopsies. This strategy, if proven effective in clinical trials, could contribute to more efficient patient management, ultimately offering a “one-stop-shop” for diagnosis and management of BCC and reducing patient anxiety, visits, and costs.

## DISCLOSURE

This work was supported by The Society of MSK and an NIH/NCI Cancer Center Support Grant (P30 CA008748). Milind Rajadhyaksha is a former employee of and owns equity in Caliber Imaging and Diagnostics. No other potential conflict of interest relevant to this article was reported.

## Notes

KEY POINTS **QUESTION:** Can PARPi-FL be used in clinics for BCC detection? **PERTINENT FINDINGS:** PARP1 is overexpressed in BCC lesions compared with nonmalignant clinical mimickers. PARPi-FL–labeled BCC can be detected through intact skin with FCM and differentiated from benign lesions. PARPi-FL can permeate skin in 2–5 min and is safe for topical application. Increasing the PARPi-FL concentration increases signal intensity within a shorter application time. **IMPLICATIONS FOR PATIENT CARE:** Our study supports PARPi-FL for clinical use to improve BCC diagnosis and management and avoid unnecessary biopsies.
